# New Famennian colonial coral (Rugosa) from the Holy Cross Mountains (Poland): an example of local evolution after Frasnian-Famennian extinction

**DOI:** 10.1007/s00114-016-1356-1

**Published:** 2016-03-16

**Authors:** Błażej Berkowski, Mikołaj K. Zapalski, Tomasz Wrzołek

**Affiliations:** Institute of Geology, Adam Mickiewicz University in Poznań, ul. Maków Polnych 16, 61-606 Poznań, Poland; Faculty of Geology, University of Warsaw, ul. Żwirki I Wigury 93, 02-089 Warszawa, Poland; Faculty of Earth Sciences, University of Silesia, Będzińska 60, 41-200 Sosnowiec, Poland

**Keywords:** Colonial Rugosa, Taxonomy, Famennian, Devonian extinction, Recovery

## Abstract

Colonial rugose corals are extremely rare in the fossil record after the Late Devonian (Frasnian-Famennian) extinction event. Here, we report a new genus and species, *Famastraea catenata*, from the late Famennian of the western part of the Holy Cross Mountains (Kowala) in Poland. Although this taxon is colonial, it displays many morphological characters very close to the typically late Famennian solitary species *Palaeosmilia aquisgranensis* (Frech, 1885), described earlier from the same locality. Hence, we postulate that *F. catenata* is derived from *P. aquisgranensis*. In contrast to other Famennian colonial rugose corals, the new taxon represents an example of local evolution within the group of so-called ‘Strunian’ corals. Consequently, we postulate that the new taxon represents a new colonial rugose fauna, which, however, did not survive the subsequent Late Devonian crisis (i.e. Hangenberg event). *F. catenata* most probably inhabited deeper water settings, possibly near the boundary between the euphotic and dysphotic zones, as inferred from many other benthic taxa described from this locality.

## Introduction

The Late Devonian mass extinction significantly affected most of the organisms living in marine shallow water environments. Rugose corals, especially colonial and large dissepimented taxa, almost disappeared from the fossil record until the late Famennian (see e.g. Poty 1984, 1999; Sorauf and Pedder 1986; Scrutton 1988; Berkowski 2001, 2002 and others). The subsequent reappearance of the rugose corals after the Frasnian-Famennian collapse was diachronic in nature and diversified (see Poty 1999; Berkowski 2002). The first significant occurrences of rugose corals are known in the *Palmatolepis rhomboidea* and *Palmatolepis marginifera* zones of the Famennian. Corals of that time were, however, solitary and relatively small, lacking interseptal dissepiments, and appeared in pelagic facies. These morphotypes of rugosans, commonly called the *Cyathaxonia* fauna (sensu Hill 1938) or laccophyllid taxa (sensu Różkowska 1969; Oliver 1992), are generally attributed to deeper water environments (Kullmann 1997). This generalization concerning their usefulness in ecological analyses was criticized by Fedorowski (1979, 1997), and indeed, the co-occurrence of small undissepimented coral taxa together with large dissepimented and/or colonial rugose corals is quite common in the fossil record (Oliver 1992; Somerville 1994; Fedorowski 1997; Scrutton 1998 and others). The term *Cyathaxonia* fauna is also difficult to define in a morphological sense (see the discussion of Wrzołek 2002 and Berkowski 2012). Additionally, Wrzołek (2002) discussed the nature of the Devonian extinctions and recoveries of this group of fossil corals, which is, however, not comparable to large dissepimented and colonial taxa of rugose corals.

While the process of recovery of the *Cyathaxonia* fauna proceeded in a more or less similar way in deeper water environments worldwide, the recovery of large dissepimented and colonial rugose taxa started later in the *Palmatolepis expansa* and *Siphonodella praesulcata* zones and was more endemic in nature. There is, however, one exception noted up to date from the middle Famennian of northern France, where relatively large solitary rugose coral *Breviphrentis superstes* was described by Denayer et al. (2012). A relatively rapid mass reappearance of large dissepimented taxa in various locations, called the ‘Strunian radiation’, was summarized and discussed by Poty (1999). The occurrences, palaeogeographic distribution as well as possible ways of migration of so-called ‘Strunian’ corals were also recently discussed by Denayer (2016). The discussion about the evolutionary patterns concerning the so-called ‘Strunian taxa’ is, however, limited and awaits a future time, when the type collections from Belgium, Germany and France are revised.

Famennian colonial rugose corals are extremely rare in the fossil record; hence, the reconstructions of their evolutionary patterns are obscure and highly speculative (see Berkowski 2001, 2002). Here, we describe for the first time a new taxon of colonial Rugosa, which originated from the strictly late Famennian group of solitary taxa belonging to the so-called Strunian corals.

## Material and methods

**Fig. 1:**
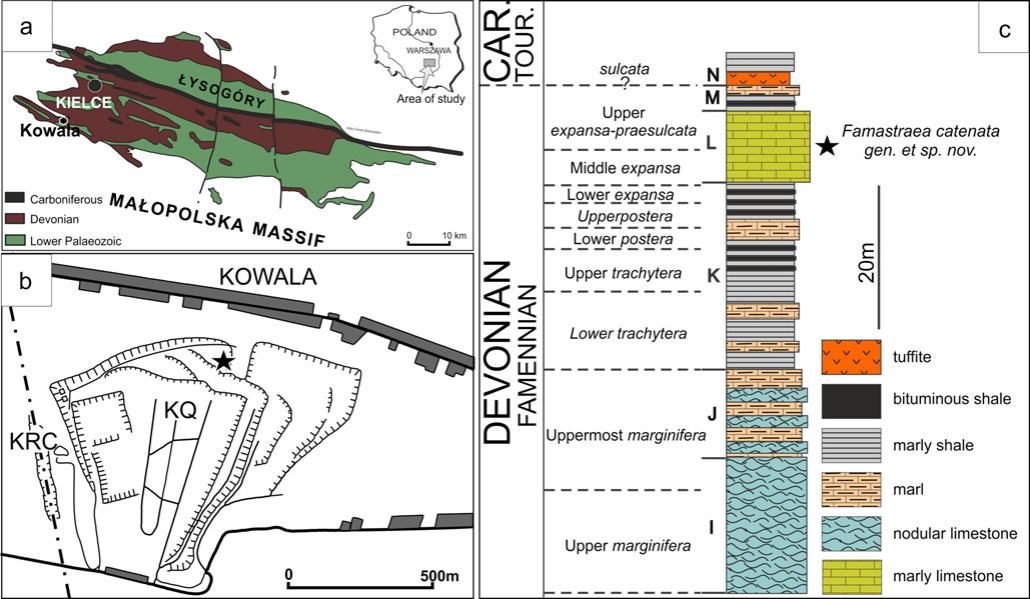
**a** Simplified geological map of the Holy Cross Mountains modified after Szulczewski (1971). Kowala locality is indicated. **b** Schematic map of the Kowala Quarry and surrounding areas: *KQ* Kowala Quarry, *KRC* Kowala railroad cut. *Star* in the *northern part* of the quarry indicates the locality where the specimens were collected (set L, upper Famennian). **c** Geological log of the middle and upper Famennian cropping out in the northern part of the Kowala Quarry. *Star* indicates the stratigraphic position of the studied specimens

The studied material comprises two colonies of rugose corals coming from the Famennian beds of the Kowala Quarry in the Holy Cross Mountains, Poland (Fig. 1). The specimens were found by the staff and students of geology from Silesia University. Thus, the material is housed at the Faculty of Earth Sciences, University of Silesia, Sosnowiec, Poland, under the abbreviation GIUS3619. Following the standard methods used for the taxonomic determinations of rugose corals, the material was studied in serial transversal and longitudinal sections (thin sections and acetate peels). The sections of the coralla were photographed and scanned using a Zeiss Stereomicroscope Discovery V20 with a Canon D70 camera, and an Epson V700 Photo scanner in the mode for transparent materials and resolution of 3200 dpi.

## Geological setting

The locality of Kowala is famous for its almost complete Upper Devonian sequence, which was first described by Szulczewski (Frasnian sets A–G; 1971) and later Berkowski (Famennian sets H–L; 1990, 1991, 2002). Szulczewski (1995) summarized the whole profile with the updated conodont zonation. Malec et al. (1995, 2014), Malec (2014), Dzik (1997, 2006), Olempska (1997) and Marynowski and Filipiak (2007) provided supplementary data on strata and fossils in the uppermost part of the sequence exposed in the trenches, cropping out in the recently exploited NE part of the quarry.

Famennian corals of the Kowala Quarry were found in two stratigraphic horizons. The lower one is the uppermost part of set J or lowermost part of set K (for the complete geological log, see Berkowski 2002). It contains a single tabulate species, which is the branching *Thamnoptychia mistiaeni*, accompanied by the heterocoral *Oligophylloides pachythecus* (described by Zapalski et al. 2016). Rugose corals are represented only by *Circellia concava*, which is a solitary coral with lonsdaleoid vesicles (Berkowski 2002). This interval is represented by knobby dark limestones intercalating with black bituminous shales, which contain blind trilobites (Berkowski 1991; Radwański et al. 2009), pyritized goniatites and locally abundant *Guerichia* bivalves (Berkowski 2002; Marynowski et al. 2010).

**Fig. 2:**
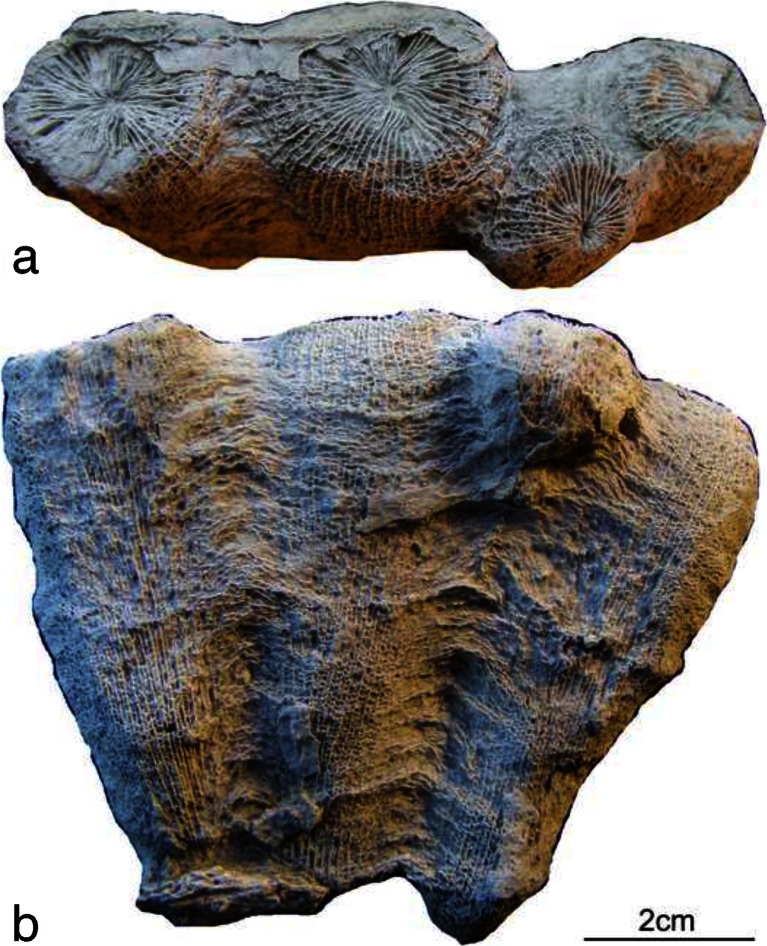
Corallum of *Famastraea catenata* gen. et sp. nov. External photographs of the holotype GIUS3619 KF014: **a** calicular view and **b** side view. Note that the offset is visible as the second juvenile corallite from the *right*

The majority of the Famennian corals of Kowala, including *Famastraea catenata* gen. et sp. nov. described in the present paper, come from the rubble of bedded pelitic and partly nodular olive-green marly limestones of set L (Fig. 2c, see also Berkowski 1991, 2002; Szulczewski 1995).

The upper part of this set of beds corresponds to the lithological set (‘complex’) A (sensu Malec et al. 1995 and Marynowski and Filipiak 2007), which is dated as the early *P. expansa* to *S. praesulcata* zones (Malec et al. 1995; Olempska 1997). Corals described so far from this horizon include scarce tabulates. Among them, Zapalski and Berkowski (2012) described ?*Yavorskia paszkowskii* and Zapalski et al. (2016) described two species of ?*Favosites*, unidentified ?alveolitid, ?*Michelinia vinni*, *Syringopora kowalensis*, *Syringopora hilarowiczi* and *Aulocystis* sp. The rugose corals were first described by Różkowska (1969) then by Berkowski (2002), who described nine species of solitary rugosans from this horizon, namely *Neaxon regulus*, *Neaxon tenuiseptatum*, *Friedbergia bipartita*, *Guerichiphyllum kowalense*, *Gorizdronia soshkinae*, *Nalivkinella rariseptata*, *Campophyllum* sp. A, ?*Spirophyllum* sp., ?*Palaeosmilia aquisgranensis* and a heterocoral *O. pachythecus*.

Apart from corals, the upper Famennian part of the sequence yielded abundant cephalopods (Czarnocki 1989; Dzik 2006; Rakociński 2006), bivalves (*Guerichia*) and brachiopods (Halamski and Baliński 2009).

## Note on the state of preservation

Both of the studied coral specimens lack an external wall (Fig. 2); hence, the external surfaces of the coralla reveal only septa and dissepiments. This phenomenon was also mentioned by Berkowski (2002) as typical for the collection of so-called Strunian solitary taxa (*Campophyllum*, ?*Palaeosmilia* and ?*Spirophyllum*) from Kowala. The lack of marginaria in the external parts of the coralla could be caused by the following factors:

1. In situ corrosion (dissolution and/or breakage) of the fragile and extremely delicate structure of the external part of the coralla (thin wall and possibly also thin transseptal, lonsdaleoid dissepiments present between the corallites in the studied colonies);
2. Possible short transport of the coralla and/or reworking, which might at least partly have mechanically worn off the external structures of coralla.

As the sediment surrounding the studied coralla is composed of marly, pelitic limestone (mudstone) intercalated by argillaceous or black bituminous shale, and lacks grainy organic detritus or any other sedimentary structures suggesting transport of the sediment, it can be inferred that the second factor should be excluded. Moreover, the environment of sedimentation in the late Famennian of Kowala seems to be relatively deep water representing dysaerobic conditions at the bottom (Berkowski 2002; Marynowski and Filipiak 2007), close to the boundary between the photic and dysphotic zones (Zapalski et al. 2016). Hence, it seems probable that the studied colonies lived in this environment, as was postulated also for the tabulate coral faunas (Zapalski et al. 2016).

## Results

### Systematic part

The new colonial coral, *F. catenata* gen. et sp. nov., is here assigned to Campophyllidae. Similarly, to the same family, we also provisionally assign the specimens described widely as *P. aquisgranensis* (Frech, 1885), due to strong affinity of this taxon with *Campophyllum* rather than with the late Viséan genus *Palaeosmilia* (see the discussion in remarks below).

Order Rugosa Milne-Edwards and Haime, 1850

Family Campophyllidae Wedekind, 1921

Emended diagnosis: solitary or colonial. Major septa are long, extending or not to the axis, straight or sinuous, sometimes carinated. Minor septa are usually contratingent, contrajuncted or contraclined. Cardinal septum is shortened in conspicuous fossula. Counter septum is as long as adjacent septa or longer. Dissepimentarium is narrow to wide, including concentric interseptal dissepiments, and herringbone and lonsdaleoid dissepiments may appear. Tabulae complete, flat or hat-shaped with downturned edges.

Genera assigned: *Campophyllum* Milne-Edwards and Haime, 1850; *Famastraea* gen. nov., and yet unrevised and unnamed genus represented by specimens traditionally assigned to *P. aquisgranensis* (Frech, 1885), occurring in the late Famennian.

#### Remarks

According to Hill (1981), the late Famennian *Campophyllum* is the sole genus representing the family Campophyllidae. In our opinion, however, several other late Famennian genera of dissepimented solitary rugose corals seem to be closely related to *Campophyllum* and should be included in the Campophyllidae. Among them, the late Famennian *P. aquisgranensis* (Frech, 1885) possesses a more advanced morphology of the corallite that seems to reveal its real affinity. Hence, it can be inferred that it is a descendant of *Campophyllum*, having very close morphological resemblance to typical Viséan *Palaeosmilia murchisoni*. These similarities and probable relationships were already presented by Berkowski (2002), who suggested that the Famennian *P. aquisgranensis* and Viséan species of *Palaeosmilia* do not represent an evolutionary lineage, as there exists a long Lower Carboniferous stratigraphic gap between their occurrences. Hence, *P. aquisgranensis* and *P. murchisoni* seems to be homeomorphs and *P. murchisoni* can be regarded as the so-called ‘Elvis taxon’ (sensu Erwin and Droser 1993). Poty (2010) additionally suggested that this distinction is also emphasized by differences in septal microstructures, but in our opinion, the differences are not clearly illustrated and may result from ecologically and/or diagenetically controlled processes.

In the rich collections of so-called Strunian corals from typical localities (Belgium, Germany and France) it is possible to trace many intermediate forms between *Campophyllum* and *P. aquisgranensis* morphologies, especially when comparing sections of adult stages of growth (E. Poty, personal communication; B. B., personal observations). These observations were also confirmed by Denayer (2016). In the collection from Kowala (see Berkowski 2002) yielding only a few specimens, these two late Famennian genera are clearly differentiable, especially tracing ontogenetic successions. One may easily identify and separate taxa (see Berkowski 2002), revealing the amplexoid early ontogeny of *Campophyllum* and zaphrentoid early ontogeny typical of *P. aquisgranensis*, which is also well emphasized in the new genus *Famastraea*.

Genus: *Famastraea* gen. nov.

Type species: *F. catenata* gen. et sp. nov.

Species assigned: monotypic.

Type horizon: Upper Devonian, upper Famennian, *P. expansa*/*S. praesulcata* zones. Lithological set L of Berkowski (1991, 2002).

Type locality: northern margin of the Kowala Quarry, Holy Cross Mountains.

Derivation of the name: combination of the last Upper Devonian stage name Famennian and Latin *astraea*—star.

Diagnosis: colonial, aphroid developing in a row (chain-like colonies); corallites large, joined to neighbouring corallites on one or both sides by lonsdaleoid dissepimentarium. In corallites, major septa are long, almost reaching axis, and arranged radially. Protosepta are as long as other major septa. Cardinal septum and alar septa may be situated in shallow and long fossulae. Minor septa are straight or slightly contraclined and at the axial end may be contrajuncted by small dissepiments. Dissepimentarium is composed of concentric to herringbone dissepiments, becoming lonsdaleoid towards periphery. Tabularium is biform; tabulae are flat, sagging in the middle and with downturned edges.

#### Remarks

**Fig. 3:**
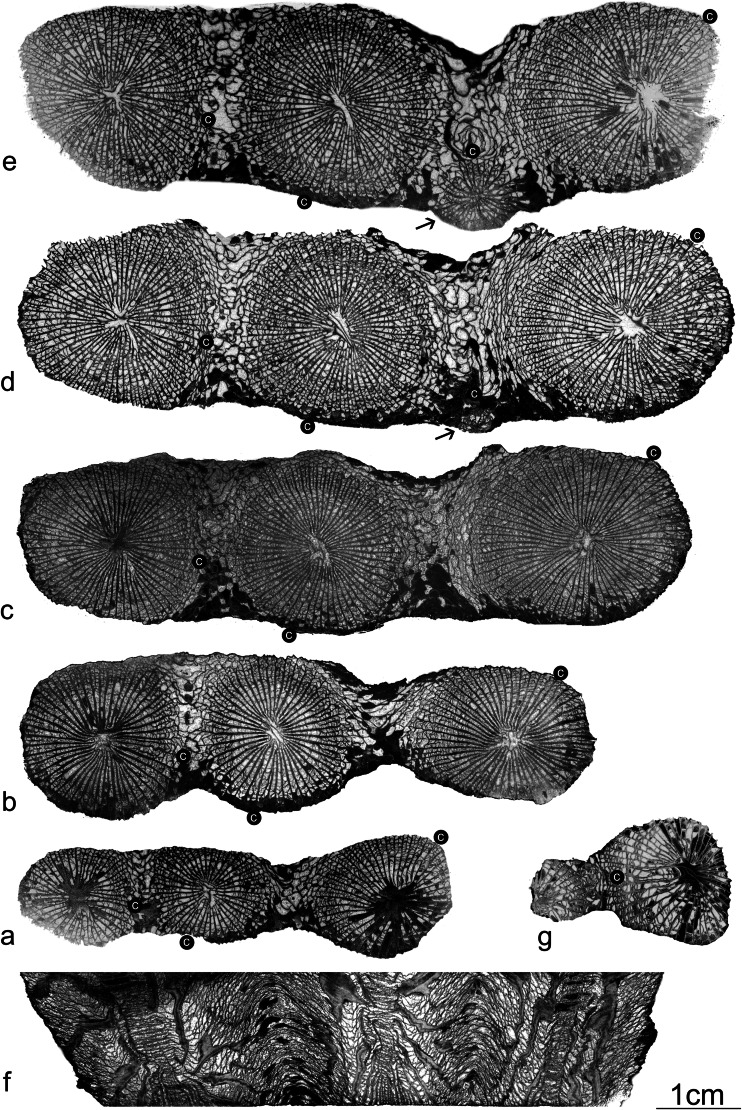
*Famastraea catenata* gen. et sp. nov. **a**–**e** Serial transversal thin sections and peels of the holotype GIUS3619 KF014, showing successive stages of astogeny and corallite ontogeny. Note that *c in black circle* indicates a cardinal septum of each corallite. *Arrows* on **d** and **e** indicate the formation of the offset. **f** Longitudinal section of the holotype GIUS3619 KF014. **g** Transversal thin section of the paratype GIUS3619 KF058. Note that *c in black circle* indicates cardinal septum

*Famastraea* gen. nov. possesses characters of the solitary genera of the *Campophyllum*-*P. aquisgranensis* lineage (especially the members representing *Palaeosmilia*-like descending taxa), which here additionally display the phenomenon of coloniality. The coralla are, however, composed of only a few, but relatively large, corallites (two to four corallites developed in a row are present in a studied material; cf. Fig. 3). On the other hand, the studied specimens show high integration of the colony, which is aphroid with some remnants of restricted walls occurring between the corallites. This character is also noted in the Viséan genus *Palastraea* that evolved from *Palaeosmilia*. Morphologically, *Famastraea* differs from *Palastraea* in possessing shorter and more amplexoid septa and a less developed concentric dissepimentarium/tabularium zone in the central part of the corallite.

*F. catenata* sp. nov.

**Fig. 4:**
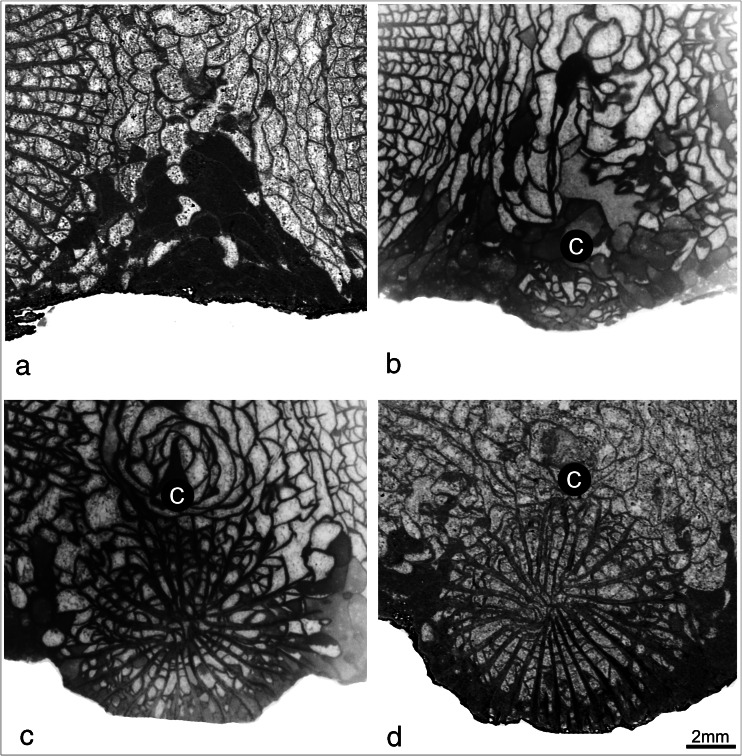
**a**–**d** Four successive blastogenetic stages of the offset in the holotype corallum GIUS/KF14. Note that *c in black circle* indicates cardinal septum

See Figs. 2, 3, 4 and 6(a, b).

Holotype: GIUS3619 KF014.

Paratype: GIUS3619 KF058.

Type horizon: Upper Devonian, upper Famennian, *P. expansa*/*S. praesulcata* zones. Lithological set L of Berkowski (1991, 2002).

Type locality: northern margin of the Kowala Quarry, Holy Cross Mountains, Poland.

Derivation of the name: after the cateniform (chain-like) shape of the corallum of the described holotype.

Diagnosis: *Famastraea* possessing 56–60 septa by 24–26 mm diameter in maturity.

Material: two specimens (small colonies). Holotype is composed of four corallites (Fig. 2). Paratype is fragmentary preserved and composed of two corallites representing mature (parent corallite) and juvenile (daughter corallite) stages (Fig. 3g).

Description of the holotype (Figs. 2, 3a–f, 4 and 6(a, b)): corallum is composed of four corallites arranged in a row, three of them are mature and one is in neanic stage, developing as an offset on the lonsdaleoid dissepiments between two mature corallites. The maximum length of the colony is 87 mm, the maximum width is 26 mm and the height measured from the bottom of the colony to the top is 72 mm. Colony lacks external wall, part of lonsdaleoid dissepimentarium and calical rims of the corallites.

In transversal sections, corallites are connected by lonsdaleoid dissepiments forming an aphroid colony. Fragmentary walls were observed between the corallites. Corallites are large, up to 25 mm in diameter, with numerous (up to 60), long, radially arranged and amplexoid major septa. Protosepta are hardly distinguishable; in most transversal sections, they are as long as other major septa but may be slightly longer, especially on the tabulae. Cardinal and alar septa may be emphasized by the presence of shallow and elongated fossulae. Orientation of cardinal-counter plane in different corallites of the corallum is random (does not reveal uniform direction). Minor septa are straight or slightly contraclined, at their ends may be contrajuncted (joined to the major neighbouring septum by small dissepiment). Distally, first minor and then major septa are disrupted by transseptal dissepiments. Dissepiments are concentric near the tabularium, herringbone towards external wall and finally lonsdaleoid in distal part and between the corallites.

In longitudinal section (Fig. 3f), tabulae are mostly flat with downturned edges, in the central zone, accompanied by additional irregularly distributed domed plates. Dissepiments in a longitudinal section are globose and steeply sloping towards the tabularium, externally become flatter and form an elevated structure between the corallites. In these places, short crests are developed rarely, representing underdeveloped fragments of the wall (Fig. 3f).

Astogeny: the early stages of astogeny (colony development) are not preserved; hence it is not possible to distinguish which (if any) of the three similarly developed corallites represents the protocorallite.

Blastogeny: increase is lateral (Fig. 4). The process of offset formation could be traced only on one corallite, which developed on lonsdaleoid dissepimentarium (see Figs. 3d, e and 4). The septal arrangement of the early stages is zaphrentoid but, in later stages, becomes more amplexoid. This is a typical character distinctive for the early ontogeny of the representatives of *P. aquisgranensis* from Kowala.

Paratype: the transversal section of the paratype (Fig. 3g) represents mature, but relatively young stage of growth of the parent and neanic stage of the offset. Both are connected by lonsdaleoid dissepiments.

Occurrence: at present, only known from type locality and horizon.

## Discussion

**Fig. 5:**
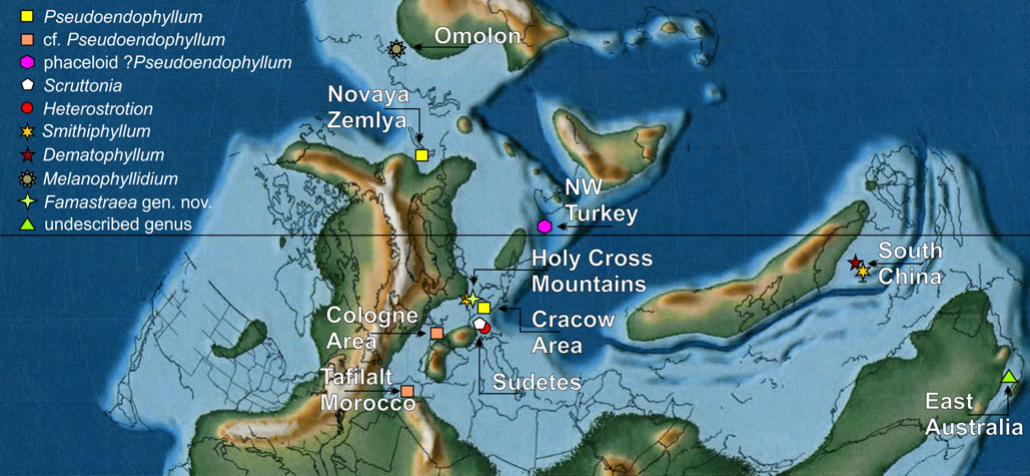
Portion of palaeogeographic map at the Devonian-Carboniferous transition (Scotese 2014). Occurrences of colonial Rugosa in the latest Famennian are indicated in appropriate localities

Famennian colonial rugose corals are extremely rare in the fossil record (see Berkowski 2001, 2002). Although present in the late Famennian, they are sparsely distributed in the world (Fig. 5), representing only several taxa, and additionally, they are not numerous (in terms of the number of specimens). Some of them, like the three Famennian species of the genus *Scruttonia*, known from the Sudetes (Poland), have highly integrated, massive thamnasteroid-aphroid coralla (Fedorowski 1991; Berkowski 2002). They clearly represent survivors of the Frasnian-Famennian extinction event and may be regarded as ‘Lazarus taxa’ (sensu Jablonski 1986). Others, like the more widely distributed genus *Pseudoendophyllum* (Gorsky 1935, 1938; Onoprienko 1979; Berkowski 2001, 2002), forming cerioid colonies, seem to be homeomorphic to the Middle Devonian genus *Endophyllum* and thus may be regarded as ‘Elvis taxa’ (sensu Erwin and Droser 1993). On the other hand, ?*Pseudoendophyllum* sp. as described recently by Denayer (2016) from NW Turkey is phaceloid, possessing a different type of septal arrangement, and in our opinion, represents rather another new genus. While *Scruttonia* disappeared from the fossil record by the end of the Famennian, *Pseudoendophyllum* probably survived and evolved worldwide in the Early Carboniferous into the *Parastelechophyllum*-*Stelechophyllum* lineage (see Berkowski 2001). Similarly, the phaceloid genus *Heterostrotion*, which appeared in the late Famennian of the Sudetes (Fedorowski 1991; Berkowski 2002), survived the subsequent Hangenberg event at the Devonian-Carboniferous boundary (Berkowski 2001) and gave rise to new species in the Early Carboniferous of the Ardennes, South China and Vietnam (Poty and Xu 1996, 1997; Khoa 1996 and others). Among the other Famennian records of colonial Rugosa, there is also one that was described but is now inaccessible, i.e. a lost cerioid colony described by Wulff (1923) from the Cologne area in Germany. This colony, and an undescribed colony from the Famennian of Tafilalt in Morocco (D. Weyer, personal communication in 2002), might also represent the genus *Pseudoendophyllum* or a related taxon. The Famennian species of *Smithiphyllum* described from Poland (Holy Cross Mountains, see Różkowska 1969) and South China (Poty 1999) as well as the Chinese genus *Dematophyllum* (Poty and Xu 1997; Poty 1999) may also represent Lazarus taxa. One bizarre colonial genus *Melanophyllidium* was also described from Omolon in eastern Siberia (Poty and Onoprienko 1984; Poty 1999). Finally, a few extraordinary colonies of Rugosa have also been found in Australia (J. Jell, personal communication in 1995), but they still remain undescribed and their relation to other genera is unknown.

**Fig. 6:**
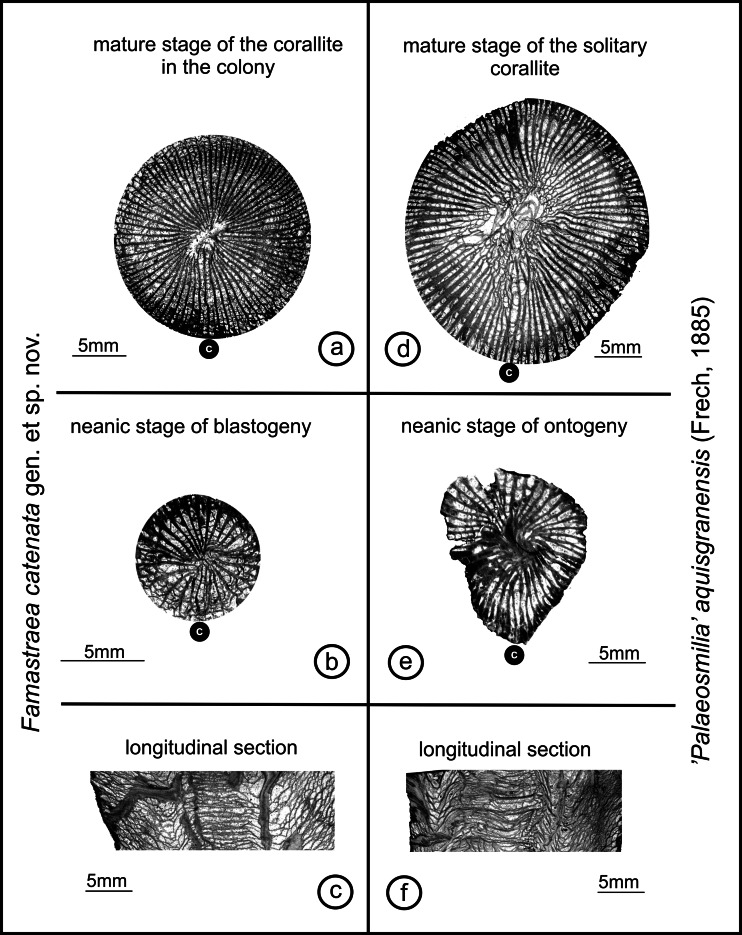
Comparisons of morphological characters in *Famastraea catenata* gen. et sp. nov. (*a*–*c* specimen GIUS3619 KF014) and *Palaeosmilia aquisgranensis* (Frech, 1885) (*d*–*f* specimen UAM Tc-B\01\03). *a* Section of the mature stage of the corallite; *b* section of the neanic stage of the offset. The sections of the corallites are cropped to a *circle* for better visibility of the important internal characters. *c* Longitudinal section of the corallite. *d* Section of the mature stage of the solitary corallite. *e* Section of the neanic stage of ontogeny. *f* Longitudinal section of the corallite. Cardinal septum is indicated by *c in black circle*

 In contrast to the taxa of colonial Rugosa described above, the new colonial genus and species *F. catenata* provides a somewhat different evolutionary example. Comparative studies of the internal morphology of the corallites of the new taxon reveal a very close affinity to the late Famennian (Strunian) solitary coral *P. aquisgranensis* from Kowala (Fig. 6). In both taxa, the structure of the septa, septal arrangement, shape of the fossula, types and number of dissepiments (see comparison on Fig. 6(a, d)) are almost identical. Both possess mostly complete tabulae (Fig. 6(c, f)) with downturned edges and display delicate invagination in the centre (hat-shaped tabulae). Moreover, the septal arrangements in the early stages of ontogeny in the solitary specimens of *P. aquisgranensis* and comparable stages of the early blastogeny (offset formation during increase) in the studied colony of *F. catenata* are also very similar (Fig. 6(b, e)). The lack of lonsdaleoid dissepiments in *P. aquisgranensis*, which are present between corallites in the colonial taxon *F. catenata*, may result in Kowala specimens from taphonomic processes, as none of the specimens have a preserved external wall. These observations, in spite of the scarcity of material, allow us to postulate that *F. catenata* may have been derived from *P. aquisgranensis* in or not far from the late Famennian shelves of the present-day Holy Cross Mountains. On the other hand, we can speculate that the ability of colony formation in these taxa might have evolved locally, only in the Holy Cross Mountains, as we do not have similar examples in the Famennian in other localities. A similar transition from the solitary into colonial form recurrently appeared much later (approx. 25 M years) in the upper Viséan as the *Palaeosmilia*-*Palastraea* lineage on a much wider scale (Rodríguez and Somerville 2007). Whether this was a true recurrent evolutionary trend developed in morphologically similar taxa or simply an example of homeomorphy is difficult to assess due to the obscure palaeontological material. But, concerning the Famennian example described here, we can trace in situ a local trend displaying an evolutionary linkage from the solitary to colonial form after the Frasnian-Famennian mass extinction.

## Conclusions

The late Famennian colonial rugose coral *F. catenata* gen. et sp. nov. is a colonial taxon of the family Campophyllidae and is recorded at present only in the Holy Cross Mountains (Poland). The structure of the corallites in the colony of *F. catenata* as well as their early stages of blastogeny reveal a close affinity to the structure and early ontogeny of the solitary taxon *P. aquisgranensis* described by Berkowski (2002) from this same locality and stratigraphic horizon. It seems that the evolution of the members of Campophyllidae in the late Famennian was endemic in nature and the appearance of colonial forms belonging to this family was restricted in space and time.
